# Pharmacokinetic Drug‐Drug Interaction Study of Omecamtiv Mecarbil With Amiodarone and Digoxin in Healthy Subjects

**DOI:** 10.1002/cpdd.1028

**Published:** 2021-10-11

**Authors:** Ashit Trivedi, Winnie Sohn, Cheng‐Pang Hsu, Pegah Jafarinasabian, Hanze Zhang, Shauna Hutton, Stephen Flach, Siddique Abbasi, Sandeep Dutta, Edward Lee

**Affiliations:** ^1^ Amgen Inc Thousand Oaks California USA; ^2^ Covance Inc Madison Wisconsin USA

**Keywords:** amiodarone, digoxin, drug‐drug interaction, omecamtiv mecarbil, P‐glycoprotein

## Abstract

Omecamtiv mecarbil (OM), a novel cardiac myosin activator, is being evaluated for the treatment of heart failure with reduced ejection fraction. In vitro studies demonstrate OM as a substrate and inhibitor of P‐glycoprotein (P‐gp), which can result in drug‐drug interactions. Two phase 1, open‐label studies assessed the effect of coadministration of OM (50‐mg single dose) on the pharmacokinetics of digoxin (0.5‐mg single dose; N = 15), a P‐gp substrate, and the effect of coadministration of amiodarone (600‐mg single dose), a P‐gp inhibitor, on the pharmacokinetics of OM (50‐mg single dose; N = 14) in healthy subjects. The ratios of the geometric least squares mean (90% confidence interval [CI]) of digoxin coadministered with OM vs digoxin alone for area under the plasma concentration–time curve (AUC) from time 0 to infinity, AUC from time 0 to the time of the last quantifiable concentration, and maximum observed plasma concentration were 1.06 (90%CI, 0.99‐1.14), 1.06 (90%CI, 0.98‐1.14), and 1.08 (90%CI, 0.92‐1.26), respectively. The ratios of the geometric least squares mean of OM coadministered with amiodarone vs OM alone for AUC from time 0 to infinity, AUC from time 0 to the time of the last quantifiable concentration, and maximum observed plasma concentration were 1.21 (90%CI, 1.08‐1.36), 1.21 (90%CI, 1.07‐1.36), and 1.08 (90%CI, 0.96‐1.22), respectively. In conclusion, OM coadministered with digoxin or amiodarone did not result in any clinically relevant pharmacokinetic drug‐drug interactions.

Omecamtiv mecarbil (OM) is a direct, small‐molecule activator of cardiac myosin and is currently under investigation for the treatment of heart failure (HF) with reduced ejection fraction. In vitro and preclinical studies have demonstrated that OM increases the contracting force of cardiac myocytes by allosterically activating the catalytic domain of myosin and increasing the number of myosin heads primed for engagement with actin filaments during systole without affecting intracellular calcium and oxygen consumption.^1^, ^2^, ^3^, ^4^, ^5^, ^6^, ^7^

Clinical studies have demonstrated that OM improves cardiac function in healthy subjects and patients with HF.^8^, ^9^ In a recently completed phase 3 study in patients with HF with reduced ejection fraction (GALACTIC‐HF), those who received OM had a lower risk of HF events and cardiovascular death than those who received placebo.^10^

OM exhibits a linear dose‐proportional pharmacokinetic (PK) profile with a median time (t_max_) of maximum observed plasma concentration (C_max_) of 2 hours and a mean apparent terminal elimination half‐life (t_1/2_) of 18.5 hours.^8^, ^11^, ^12^, ^13^ OM is primarily metabolized in humans by the cytochrome P450 (CYP) enzymes CYP3A4 and CYP2D6 to the M3 and M4 circulating metabolites, which are significantly less potent than OM.^14^, ^15^, ^16^ In addition, hepatic impairment, renal impairment, or hemodialysis does not significantly affect the PK profile of OM.^15^, ^17^

Patients treated for HF generally have comorbidities and require concomitant medications. This has the potential to cause PK drug‐drug interactions (DDIs) that may lead to untoward clinical effects. P‐glycoprotein (P‐gp) is a transmembrane protein that pumps xenobiotics out of cells and plays an important role in the clearance mechanism for some drugs.^18^ Digoxin is a cardiac glycoside and a well‐established, US Food and Drug Administration (FDA)‐recognized P‐gp substrate that has been used to assess the inhibitory or activating potential of other concomitant drugs.^19^, ^20^ Amiodarone is a small‐molecule, antiarrhythmic medication indicated for the treatment of recurrent ventricular fibrillation and recurrent hemodynamically unstable ventricular tachycardia and is an FDA‐recognized P‐gp inhibitor for use in DDI studies. Amiodarone thus reduces the clearance of and increases exposure to digoxin, similar to that observed with other P‐gp inhibitors. Amiodarone is also a strong inhibitor of CYP3A4 and can, therefore, lead to CYP3A4‐mediated DDIs.

In vitro studies have shown that OM is both an inhibitor and a substrate of P‐gp (half maximal inhibitory concentration = 2.29 ± 1.25 μM; inhibition constant = 1.73 μM). Following a PK‐based dose titration, the anticipated OM plasma concentration in patients with HF is between 200 and 750 ng/mL (>0.5 μM); therefore, because the FDA recommends an in vitro DDI study for a new chemical entity if the [I]/inhibition constant >0.1, we explored for a potential clinical drug interaction between OM and commonly administered concomitant medications, such as digoxin or amiodarone. Herein, we investigated the effect of OM on the PK of digoxin and the effect of amiodarone on the PK of OM.

## Methods

### Study Design

**Figure 1:**
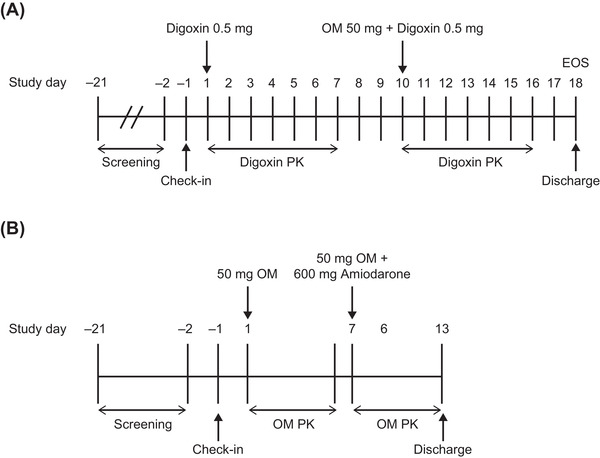
Study designs for (A) OM‐digoxin and (B) OM‐amiodarone. EOS, end of study; OM, omecamtiv mecarbil; PK, pharmacokinetics.

Two distinct phase 1, open‐label, single‐center, fixed‐sequence studies were conducted in healthy subjects to evaluate the PK DDI potential of OM when coadministered with digoxin or amiodarone (Figure 1A,B). The OM‐digoxin and OM‐amiodarone studies were conducted at Covance Clinical Research Unit, Inc in Madison, Wisconsin, and Daytona Beach, Florida, respectively. Screening for eligibility was performed within 21 days before administration of the first dose. One day before study initiation (day –1), subjects were admitted into and confined to the Clinical Research Unit until the end of the study on day 18 for the OM‐digoxin study or day 13 for the OM‐amiodarone study. Blood and urine samples were collected at prespecified time points to measure the plasma concentrations of digoxin or OM. Safety and tolerability were monitored throughout the study. The study protocol, trial activities, and informed consent form were reviewed and approved by the Salus Institutional Review Board, Austin, Texas. All subjects in the study provided written informed consent before study enrollment and had the option to withdraw at any time during the study. The study was conducted in accordance with the principles derived from the Declaration of Helsinki, International Ethical Guidelines of the Council for International Organizations of Medical Sciences, Good Clinical Practice Guidelines of the International Conference for Harmonisation, and relevant regulatory requirements.

#### OM‐Digoxin Study

This study evaluated the effect of OM on the PK of digoxin. On days 1 and 10, subjects received a single oral dose of 0.5‐mg digoxin (2 × 0.25‐mg tablets) alone and with a single oral dose of a 50‐mg OM modified‐release (MR) tablet, respectively, after an overnight fast (≥10 hours). Subjects continued fasting for at least 4 hours after dosing on days 1 and 10.

#### OM‐Amiodarone Study

This study assessed the effect of amiodarone on the PK of OM. On day 1, subjects received a single oral dose of a 50‐mg OM MR tablet after an overnight fast (≥10 hours). On day 7, subjects received a single oral dose of a 50‐mg OM MR tablet with a single oral dose of 600‐mg amiodarone (3 × 200‐mg tablets) after an overnight fast (≥10 hours).

### Study Subjects

For both studies, subjects aged 18 to 55 years with a body mass index between 18 and 30 kg/m^2^ were screened for eligibility. Subjects had to be in good health as assessed by medical history of clinically significant findings, physical examination, 12‐lead electrocardiogram, vital signs measurements, and laboratory evaluations as assessed by the investigator.

#### OM‐Digoxin Study

Fifteen subjects enrolled in the study, of whom 14 proceeded to completion. One subject discontinued the study due to behavioral issues. The mean age of the subjects was 34.5 (standard deviation [SD], 7.6) years, 8 (53.3%) were men, and 9 (60.0%) were White. Baseline demographics and clinical characteristics are shown in Table 1.

**Table 1:** Summary of Baseline Demographics and Clinical Characteristics

Parameter	OM‐Digoxin Study (N = 15)	OM‐Amiodarone Study (N = 14)
Age, y, mean (SD) Median (min‐max)	34.5 (7.6) 33.0 (23‐47)	37.6 (10.7) 41.0 (18‐54)
Sex, male, n (%)	8 (53.3)	13 (92.9)
BMI, kg/m^2^, mean (SD) Median (min‐max)	25.0 (2.9) 25.0 (20.2‐29.4)	24.8 (3.7) 26.0 (19.2‐29.9)
Height, cm, mean (SD) Median (min‐max)	172.3 (12.0) 170.3 (153.7‐193.7)	171.7 (5.0) 169.7 (165.4‐180.0)
Weight, kg, mean (SD) Median (min‐max)	74.5 (13.9) 77.5 (54.1‐99.5)	73.0 (11.4) 74.3 (57.1‐92.0)
Ethnicity, n (%)		
Hispanic or Latino	1 (6.7)	5 (35.7)
Not Hispanic or Latino	14 (93.3)	9 (64.3)
Race, n (%)		
Black or African American	5 (33.3)	5 (35.7)
White	9 (60.0)	8 (57.1)

BMI, body mass index; OM, omecamtiv mecarbil; SD, standard deviation.

#### OM‐Amiodarone Study

Fourteen subjects enrolled in the study, of whom 13 completed the study. One subject was lost to follow‐up. The mean age was 37.6 (SD, 10.7) years, 13 (92.9%) were men, and 8 (57.1%) were White. Baseline demographics and clinical characteristics are shown in Table 1.

### Pharmacokinetic Sampling

#### OM‐Digoxin Study

Blood samples for determination of plasma digoxin concentrations were collected from each subject before dosing and the following postdose times: 0.5 to 3 hours (every 30 minutes), 4 to 12 hours (every 2 hours), 24 to 144 hours (every 48 hours) after each administration of digoxin on days 1 and 10. Urine samples for determination of urine digoxin concentrations were collected from 0 to 6, 6 to 12, 12 to 24, 24 to 48, 48 to 72, 72 to 96, 96 to 120, and 120 to 144 hours after dosing following each administration of digoxin on days 1 and 10.

A fully validated liquid chromatography–tandem mass spectrometry (LC‐MS/MS) method was used for the quantification of digoxin in human K_2_‐ethylenediaminetetraacetic acid plasma. A nominal digoxin quantitation range of 10 to 10,000 pg/mL was chosen to quantitate the samples. Samples were kept frozen at –25°C ± 5°C before analysis. A 150‐μL sample aliquot was fortified with 20 μL of internal standard (digoxin‐d_3_) working solution. Analytes were isolated using a SLE+ supported liquid extraction 96‐well plate. The eluate was evaporated under a nitrogen stream at ≈45°C and the remaining residue was reconstituted. The final extract analytes were separated on a Javelin BDS HypersilC18 loading column (2.1 × 20 mm, 5 μm; Thermo Scientific, Waltham, Massachusetts) and a Pursuit XRs 3 C18 analytical column (2.0 × 50 mm, 3 μm; Agilent Technologies, Santa Clara, California) using gradient elution with mobile phase A consisting of 5 mM ammonium acetate and mobile phase B consisting of acetonitrile:methanol:water:ammonium acetate 1M (1400:500:100:10 v/v). Separated analytes were analyzed via high‐performance liquid chromatography with tandem mass spectrometry detection using positive ion electrospray. A linear, 1/concentration^2^ weighted, least squares regression algorithm was used to quantitate samples. The m/z values for digoxin were 798.5 (Q1) and 651.2 (Q3), and for digoxin‐d_3_ were 801.5 (Q1) and 654.2 (Q3). During sample analysis for these studies, the interday precision of the quality control samples for digoxin was ≤3.15%. The interday accuracies for the OM quality control samples across studies ranged between –1.17% and 2.28%.

#### OM‐Amiodarone Study

Blood samples for determination of plasma concentrations of OM were collected before dosing; at 30 minutes; and at 1, 1.5, 2, 3, 4, 6, 8, 10, 12, 24, 48, 72, 96, 120, and 144 hours after dosing following administration of OM on days 1 and 7.

The LC‐MS/MS methods for the quantitation of OM in human K_3_‐ethylenediaminetetraacetic acid plasma were fully validated and used unique stable isotope‐labeled internal standards. The calibration curve ranges were 1.00 to 500 ng/mL for OM. Samples were prepared by first spiking a 100‐μL plasma aliquot with 20 μL of internal standard solution (500 ng/mL of D_3_‐OM). Solid phase extraction was performed using Oasis MCX 30‐mg 96‐well plates (Waters Corp, Milford, Massachusetts), and 600 μL of 10:40 ammonium hydroxide/methanol was used to elute the analytes. After drying the eluate under a nitrogen stream at ≈50°C, the residue was reconstituted with 250 μL of 70:30 mobile phase A/methanol (v/v). The extract was injected onto the LC‐MS/MS system, and the analytes were separated on a Kinetex PFP 30 × 3.00 mm, 2.6‐μm column (Phenomenex, Torrance, California) using gradient elution with mobile phase A consisting of 10:90 methanol:10 mM ammonium acetate (v/v; pH 6.0) and methanol as mobile phase B. Details of the mass spectrometry and analysis have been previously reported.^21^

### Pharmacokinetic Parameters and Safety Evaluation

The PK parameters: t_max_, C_max_, area under the plasma concentration–time curve (AUC) from time 0 to the time of the last quantifiable concentration (AUC_last_), AUC from time 0 to infinity (AUC_inf_), t_1/2_, and apparent total plasma clearance (CL/F) were determined for digoxin and OM. For digoxin, AUC from time 0 to 144 hours after dosing was determined in plasma, and the following were determined in urine: amount of drug excreted in urine from time 0 to 144 hours after dosing, renal clearance, and percentage of dose excreted in urine from time 0 to 144 hours after dosing. All concentrations below the lower limit of quantification were set to 0 for the purpose of calculating descriptive statistics. PK parameters were determined from plasma and urine samples by a validated analytical procedure with noncompartmental methods performed using Phoenix WinNonlin Version 8.1 (Certara, Princeton, New Jersey). Secondary end points included adverse events (AEs), clinical laboratory tests, 12‐lead electrocardiograms, and vital signs (supine blood pressure, supine heart rate, respiratory rate, and oral body temperature).

### Statistical Analysis

Sample size estimation for both studies was based on precedent set by other similar PK studies and not on power calculations. Up to 14 subjects were enrolled to ensure 12 subjects completed the study. A linear mixed model was applied to analyze log‐transformed PK parameters (AUC_inf_, AUC_last_, and C_max_). The model assumed a fixed effect for treatment and a random effect for subject. Estimates of geometric least squares mean (GLSM) ratios (test/reference) with corresponding 90% confidence intervals (CIs) were determined. The “test” treatments were OM with digoxin or amiodarone, and the “reference” treatments were digoxin alone or OM alone. The statistical analyses were performed using SAS Enterprise Guide Version 7.13 (SAS Institute Inc, Cary, North Carolina).

AEs were summarized using descriptive statistics and coded using the Medical Dictionary for Regulatory Activities. Data for clinical laboratory tests, 12‐lead electrocardiograms, and vital signs were summarized.

## Results

### Pharmacokinetic Analyses

#### Effect of OM on the PK of Digoxin

**Figure 2:**
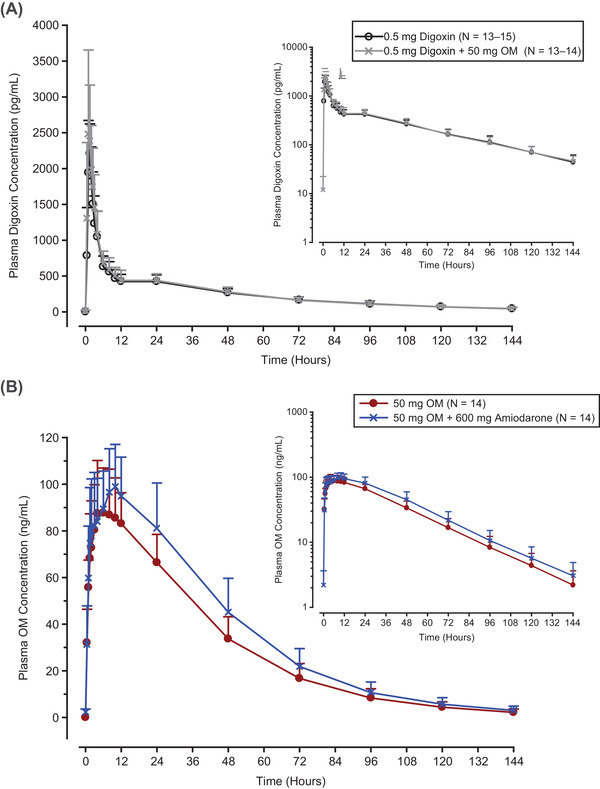
Arithmetic mean (SD) plasma concentration‐time profiles for (A) digoxin after a single oral dose of 0.5 mg digoxin alone (black line with black open circle) and in combination with 50 mg OM MR (gray line with gray “X” symbol) and (B) OM after a single oral dose of 50 mg OM MR alone (maroon line with maroon closed circle) and in combination with 600‐mg amiodarone (blue line with blue “X” symbol). Graphs are in linear scale, and inlet graphs are in semilogarithmic scale. MR, modified release; OM, omecamtiv mecarbil; SD, standard deviation.

The arithmetic mean (SD) and individual plasma concentration−time profiles for a single oral dose of 0.5‐mg digoxin alone and digoxin coadministered with a single oral dose of 50‐mg OM MR were similar (Figure 2A, Figure S1A, Figure S1B). The median t_max_ of digoxin after administration of digoxin alone was 1.5 (range, 1.0‐2.5) hours compared with 1.0 (range, 1.0‐2.0) hour after coadministration of digoxin with OM (Table 2). The arithmetic mean (SD) values for C_max_, AUC, t_1/2_, and CL/F were similar for digoxin alone and digoxin with OM (Table 2). The ratios of the GLSM (90%CI) of digoxin coadministered with OM vs digoxin alone for AUC_inf_, AUC_last_, and C_max_ were 1.06 (range, 0.99‐1.14), 1.06 (range, 0.98‐1.14), and 1.08 (range, 0.92‐1.26), respectively (Table 2).

**Table 2:** Summary of Pharmacokinetic Parameters for OM‐Digoxin Study

Study	OM‐Digoxin	
Analyte	**Digoxin**	
Parameter, Unit	**0.5 mg Digoxin (N = 15)**	**50 mg OM + 0.5 mg Digoxin (N = 14)**
t_max_, h	1.5 (1.0–2.5)	1.0 (1.0–2.0)
C_max_, pg/mL	2390 (542)	2670 (1110)
C_max_, pg/mL	2330 (1280‐3540)	2430 (1480‐5990)
AUC_last_, pg • h/mL	35 900 (7440)	38 200 (9270)
AUC_inf_, pg • h/mL	38 400 (8130)	40 800 (9460)
AUC_0–144_, pg • h/mL	35 900 (7440)	38 200 (9270)
t_1/2_, h	37.2 (6.22)	38.6 (8.83)
CL/F, L/h	13.7 (3.46)	12.9 (3.13)
Ae_0–144_, mg	0.255 (0.0403)	0.269 (0.0438)
CL_R_, L/h	7.21 (0.855)	7.24 (1.04)
F_e0–144_, %	50.9 (8.05)	53.9 (8.76)
GLSM ratio (90%CI): 50‐mg OM + 0.5‐mg digoxin (test)/0.5‐mg digoxin (reference)		
AUC_inf_, pg • h/mL	1.06 (0.99‐1.14)	
AUC_last_, pg • h/mL	1.06 (0.98‐1.14)	
C_max_, pg/mL	1.08 (0.92‐1.26)	

Ae_0–144,_ amount of drug excreted in urine from time 0 to 144 hours postdose; AUC_0–144_, area under the plasma concentration‐time curve from time 0 to 144 hours after dosing; AUC_inf_, area under the plasma concentration–time curve from time 0 to infinity; AUC_last_, area under the plasma concentration–time curve from time 0 to the time of the last quantifiable concentration; CI, confidence interval; CL/F, apparent total plasma clearance; CL_R_, renal clearance; C_max_, maximum observed plasma concentration; F_e0–144_, percentage of dose excreted in urine from time 0 to 144 hours after dosing; GLSM, geometric least squares mean; N, number of subjects with observed data; OM, omecamtiv mecarbil; SD, standard deviation; t_1/2_, apparent terminal elimination half‐life; t_max_, time of maximum observed plasma concentration.

Data are presented as arithmetic mean (SD) and reported to 3 significant figures except for t_max_, which is presented as median (range) and reported to 2 significant figures. C_max_ is also reported as median (range).

The arithmetic mean percentage of dose excreted in urine from time 0 to 144 hours after dosing of digoxin alone and digoxin with OM was 50.9% (SD, 8.05%) and 53.9% (SD, 8.76%), respectively, indicating no effect of OM on the renal elimination of digoxin (Table 2). The arithmetic mean renal clearance and amount of drug excreted in urine from time 0 to 144 hours after dosing values were also similar for digoxin alone (7.21 [SD, 0.855] L/h and 0.255 [SD, 0.0403] mg, respectively) and digoxin with OM (7.24 [SD, 1.04] L/h and 0.269 [SD, 0.0438] mg, respectively) (Table 2).

#### Effect of Amiodarone on the PK of OM

The arithmetic mean (SD) and individual plasma concentration−time profiles for a single oral dose of 50‐mg OM MR alone and OM MR with a single oral dose of 600‐mg amiodarone were characterized by similar rates of absorption, although OM MR with amiodarone exhibited a longer absorption phase with greater maximum exposures achieved relative to OM MR alone (Figure 2B, Figure S1C, Figure S1D). The elimination phases of both profiles were similar (Figure 2B). The median t_max_ of OM after administration of OM alone was 5.0 (range, 1.5‐12.1) hours compared with 8.0 (range, 1.0‐12.0) hours after coadministration of OM with amiodarone (Table 3). Arithmetic mean (SD) values for C_max_ and t_1/2_ following administration of OM alone and OM with amiodarone were similar (Table 3). OM appeared to decline in a generally biphasic manner after reaching C_max_. The ratios of the GLSM of OM coadministered with amiodarone vs OM alone for AUC_inf_, AUC_last_, and C_max_ were 1.21 (90%CI, 1.08‐1.36), 1.21 (90%CI, 1.07‐1.36), and 1.08 (90%CI, 0.96‐1.22), respectively (Table 3). Coadministration of OM with amiodarone resulted in a reduction in the arithmetic mean CL/F by ≈17% compared with OM alone (Table 3).

**Table 3:** Summary of Pharmacokinetic Parameters for OM‐Amiodarone Study

Study	OM‐Amiodarone	
Analyte	**OM**	
Parameter, Unit	**50‐mg OM (N = 14)**	**600‐mg Amiodarone + 50‐mg OM (N = 14)**
t_max_, h	5.0 (1.5–12)	8.0 (1.0–12)
C_max_, ng/mL	100 (22.6)	107 (19.6)
C_max_, ng/mL	101 (69.2‐149)	104 (80.1‐139)
AUC_last_, ng • h/mL	4180 (769)	5080 (1140)
AUC_inf_, ng • h/mL	4270 (809)	5200 (1200)
t_1/2_, h	23.7 (3.84)	24.5 (3.54)
CL/F, L/h	12.1 (2.51)	10.1 (2.33)
GLSM ratio (90%CI): 50‐mg OM + 600‐mg amiodarone (test)/50‐mg OM (reference)		
AUC_inf_, ng • h/mL	1.21 (1.08‐1.36)	
AUC_last_, ng • h/mL	1.21 (1.07‐1.36)	
C_max_, ng/mL	1.08 (0.96‐1.22)	

AUC_inf_, area under the plasma concentration–time curve from time 0 to infinity; AUC_last_, area under the plasma concentration–time curve from time 0 to the time of the last quantifiable concentration; CI, confidence interval; CL/F, apparent total plasma clearance; C_max_, maximum observed plasma concentration; GLSM, geometric least squares mean; N, number of subjects with observed data; OM, omecamtiv mecarbil; SD, standard deviation; t_1/2_, apparent terminal elimination half‐life; t_max_, time of maximum observed plasma concentration.

Data are presented as arithmetic mean (SD) and reported to 3 significant figures except for t_max_, which is presented as median (range) and reported to 2 significant figures. C_max_ is also reported as median (range).

### Safety Evaluation

All AEs were mild in severity and resolved before the end of either study. For the OM‐digoxin study, 6 subjects (40.0%) reported 8 treatment‐emergent AEs (TEAEs) after administration of digoxin alone compared with 1 TEAE in 1 subject (7.1%) after coadministration of digoxin with OM MR. For the OM‐amiodarone study, 2 subjects (14.3%) reported 2 TEAEs after administration of OM MR alone compared with 5 TEAEs in 2 subjects (14.3%) after coadministration of OM MR with amiodarone. There were no serious AEs or TEAEs leading to study discontinuation for both studies.

## Discussion

Herein, we described the outcomes of 2 clinical DDI studies evaluating the DDI potential between OM and digoxin or amiodarone. We investigated the capability of OM to inhibit P‐gp–mediated transport of digoxin, which is a commonly administered medication in patients with HF and a well‐established P‐gp substrate. Digoxin, the victim drug, was administered and evaluated at a dose of 0.5 mg, given that this is a widely used dose for digoxin in DDI assessment studies with P‐gp. OM was administered at a single dose of 50 mg, which was the highest dose strength used in phase 2 and 3 studies and was expected to be well tolerated. Multiple doses of OM were not considered in the digoxin DDI study because of safety concerns. OM dose is individualized in the clinical settings using PK‐based titration to avoid excessive OM exposures. Therefore, a single 50‐mg OM dose was selected and was considered sufficient to cover the full‐time course of the victim (digoxin) exposure and evaluate the DDI potential between digoxin and OM.

Coadministration of digoxin with OM did not result in a clinically significant DDI. The 90%CIs of the GLSM ratios of digoxin coadministered with OM vs digoxin alone for AUC_inf_, AUC_last_, and C_max_ were within the nonclinically significant DDI range of 0.8 to 1.25, except for the upper limit for C_max_, which was 1.26. The concentration of digoxin in urine was measured because digoxin is eliminated primarily unchanged by the kidneys.^22^ The percentage of the digoxin dose excreted in urine and the renal clearance were also similar when digoxin was administered alone or when coadministered with OM, further demonstrating the lack of DDI potential between digoxin and OM.

Amiodarone is also a commonly administered medication in patients with HF and a known inhibitor of P‐gp; therefore, we tested the effect of amiodarone on the PK of OM, which can also act as a P‐gp substrate in vitro. Amiodarone was administered as a single oral dose of 600 mg, which is a commonly used dose in DDI assessment studies with P‐gp. OM was also administered at the highest clinical single dose of 50 mg for the same reasons described previously. As per FDA guidance, a single‐dose regimen was evaluated because it was considered more sensitive in measuring PK changes than a multiple‐dose regimen.

Amiodarone has a rapid absorption with a t_max_ of 3 to 7 hours and a biphasic elimination with an initial one‐half reduction of plasma levels after 2.5 to 10 days. It also shows extensive accumulation in various sites including highly perfused organs, such as the liver, which implies slow elimination from the liver. Therefore, the systemic and hepatic levels of amiodarone are expected to be high during the first 2 to 3 days after coadministration. As the majority of OM is eliminated in 2 to 3 days after administration, the single‐dose design provided sufficient exposures to both victim and perpetrator to evaluate the involvement of P‐gp in OM‐amiodarone interactions primarily due to intestinal absorption and hepatic elimination. An analysis of the effect of a single dose of 600‐mg amiodarone on the PK of OM in the present study suggested a minimal but nonclinically meaningful DDI interaction between OM and amiodarone. The PK profile for OM when coadministered with amiodarone was characterized by a longer absorption phase compared with OM alone. The CL/F value for OM was also lower when OM was coadministered with amiodarone compared with OM alone. However, C_max_ and t_1/2_ were similar, whether OM was administered alone or coadministered with amiodarone.

There are a few limitations to the OM‐amiodarone DDI study. A single dose of amiodarone was administered in this study. It should be noted that if the DDI potential of an inhibitory drug is being investigated, as is in the case of amiodarone acting as a P‐gp inhibitor, it may be prudent to further evaluate multiple (vs single) dose administrations of amiodarone to investigate the impact of a worst‐case scenario of steady‐state amiodarone concentrations on OM PK and confirm the results observed in the current clinical study. Multiple doses of amiodarone may lead to alternative outcomes for OM exposures due to DDI interaction at a longer duration of amiodarone exposures.

In phase 3 trials (GALACTIC‐HF), OM was administered as 25 mg, 37.5 mg, or 50 mg twice daily following a PK‐based dose titration in patients with HF. Excessive plasma OM concentrations (>1200 ng/mL) were associated with signs and symptoms of myocardial ischemia. The PK‐based titration was employed to maintain OM plasma concentrations between 200 and 750 ng/mL at a steady state. On coadministration of amiodarone with OM, the OM plasma concentrations are expected to remain within the therapeutic concentration window. Hence, no clinically relevant DDI effect between OM and digoxin is anticipated in patients with HF, and no dose adjustments are required for the coadministration of OM with amiodarone in patients with HF.

## Conclusions

Coadministration of OM with digoxin or amiodarone was not associated with any clinically relevant PK DDIs. Overall, OM had a favorable safety profile when administered alone or in combination with digoxin or amiodarone.

## Conflicts of Interest

A.T., W.S., C.‐P.H., P.J., H.Z., S.H., S.A., S.D., and E.L. are employees and shareholders of Amgen Inc. S.F. has no relevant funding or compensation to disclose.

## Data Sharing Statement

Qualified researchers may request data from Amgen clinical studies; complete details are available at http://www.amgen.com/datasharing.

## Author Contributions

A.T., S.D., and E.L. participated in the conception and planning of the study; A.T., P.J., H.Z., S.F., S.D., and E.L. participated in the design of the study. All authors participated in the acquisition, analysis, and/or interpretation of data. All authors critically revised and approved the final version of the article.

## Funding

This study was funded by Amgen Inc.

## Supporting information

Supporting Information.

Supporting Information.
