# Supplementary figures and images for: Pharmacokinetic Drug‐Drug Interaction Study of Omecamtiv Mecarbil With Amiodarone and Digoxin in Healthy Subjects
Source: Clin Pharmacol Drug Dev. 2021 Oct 11;11(3):388–96. doi: 10.1002/cpdd.1028 (PMC9293137; doi:10.1002/cpdd.1028)

**Figure S1**  
**(A)**

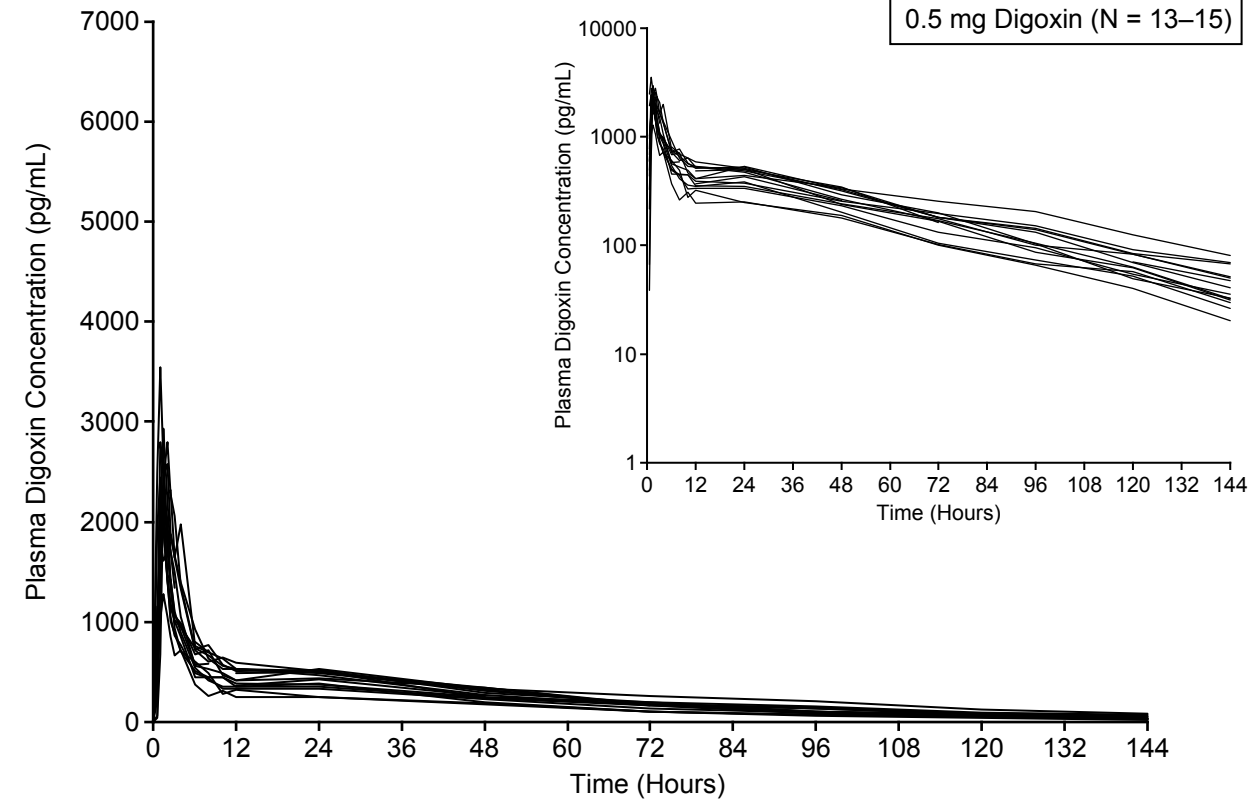

**Figure S1**  
**(B)**

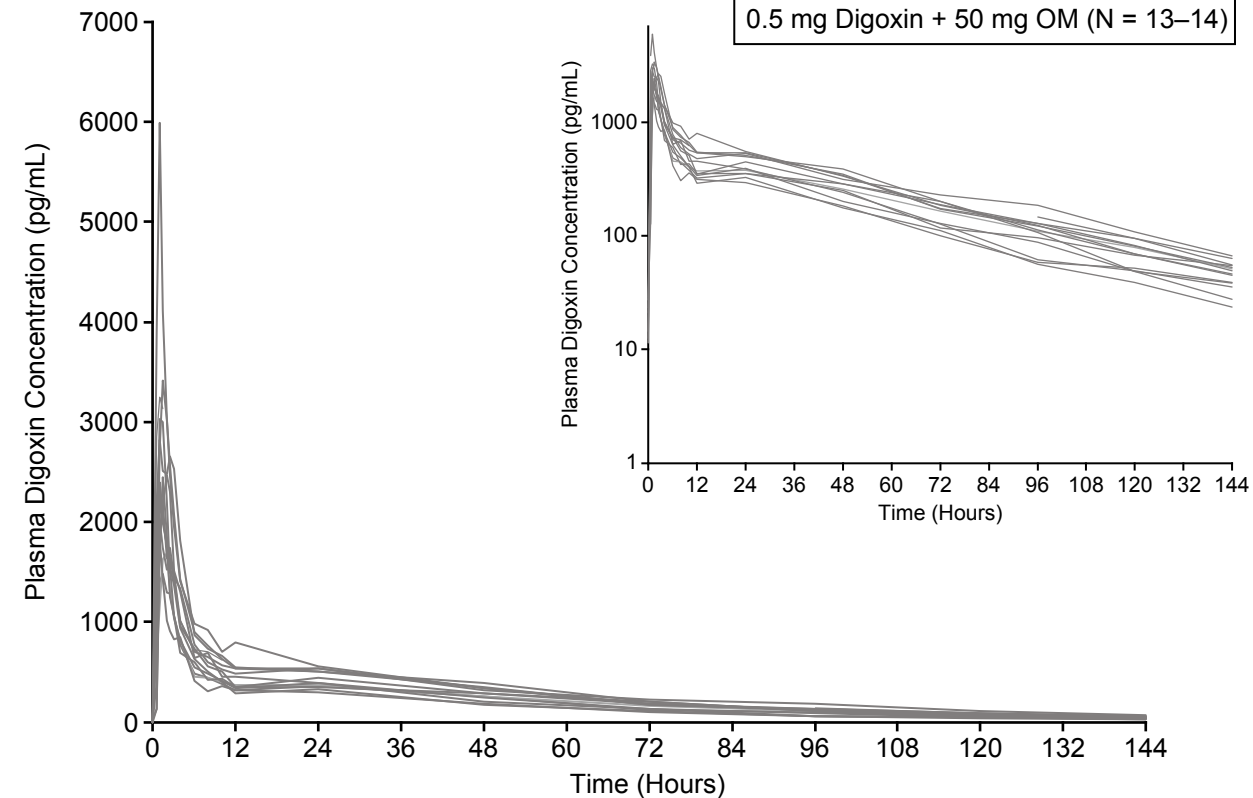

**Figure S1**  
**(C)**

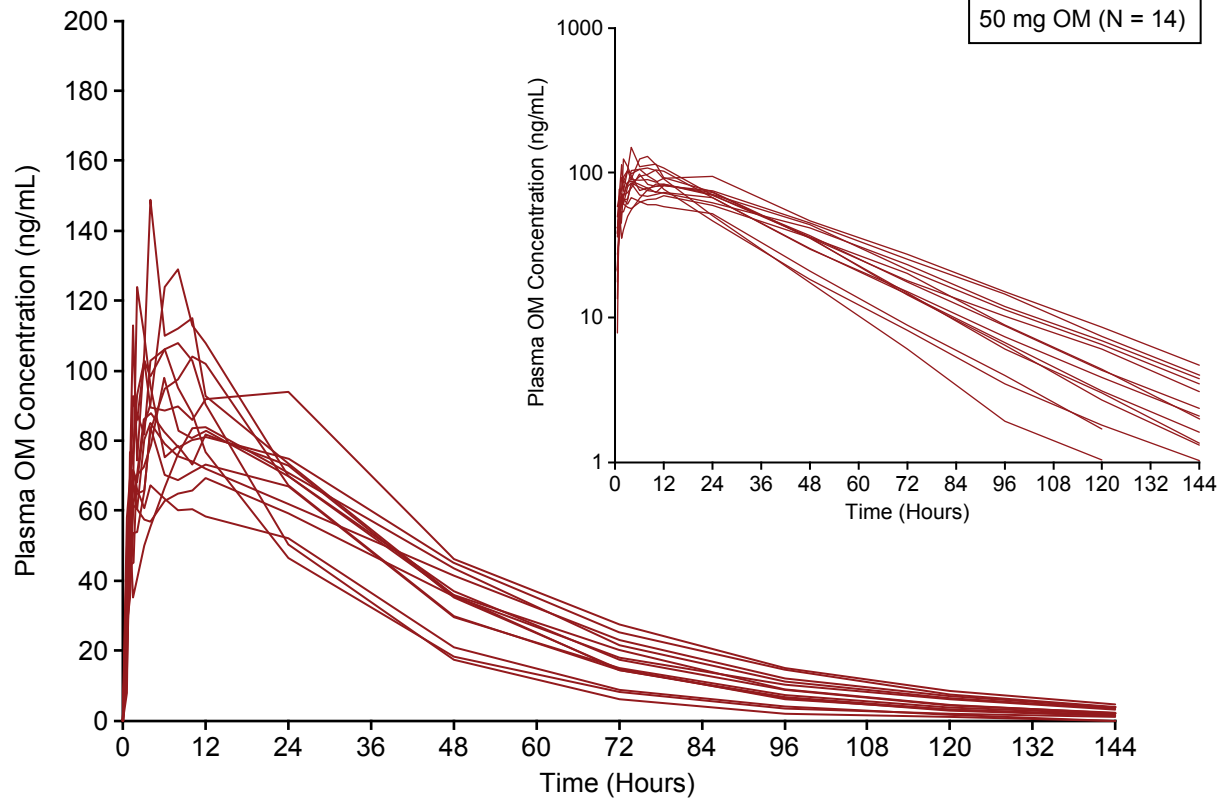

**Figure S1**  
**(D)**

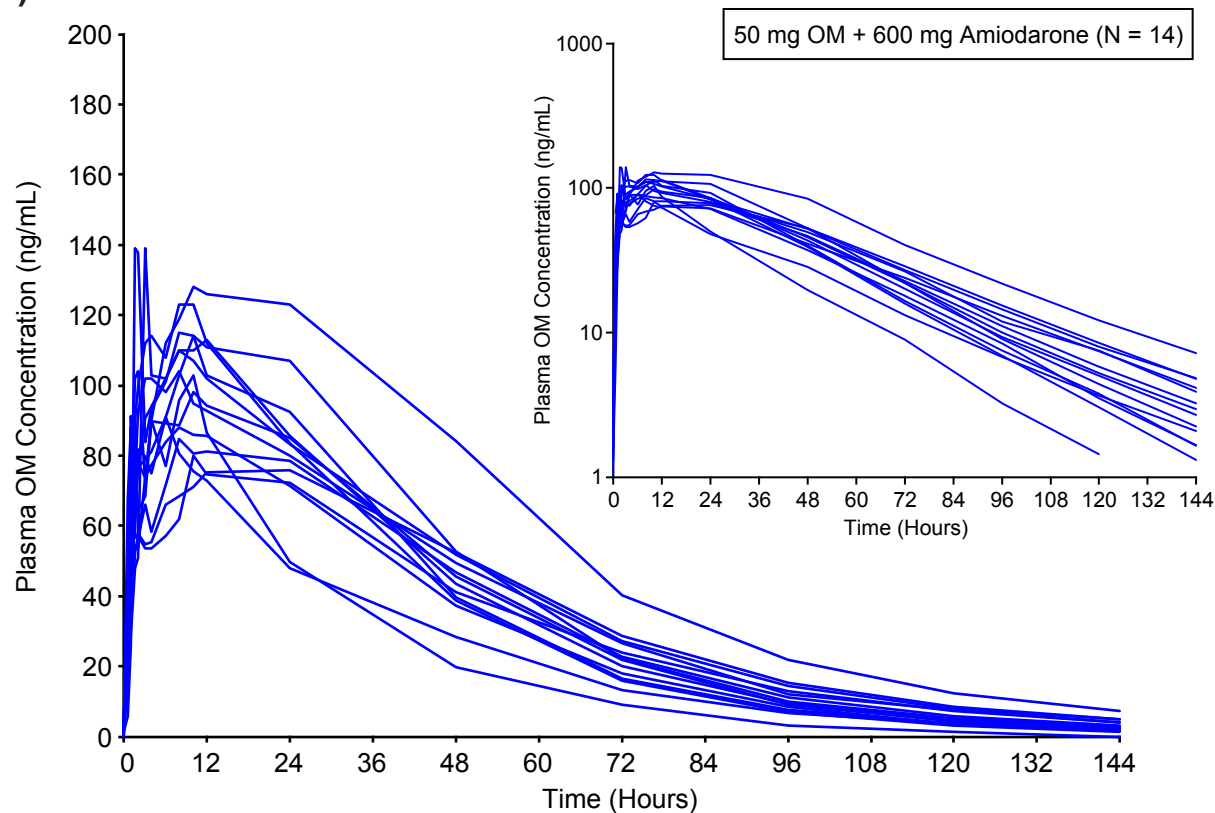

Supplement: Supplementary file 1 — Supporting Information. [file CPDD-11-388-s002.pdf]
