# Supplementary material for: Pharmacokinetic Drug‐Drug Interaction Study of Omecamtiv Mecarbil With Amiodarone and Digoxin in Healthy Subjects
Source: Clin Pharmacol Drug Dev. 2021 Oct 11;11(3):388–96. doi: 10.1002/cpdd.1028 (PMC9293137; doi:10.1002/cpdd.1028)
Supplement: Supplementary file 2 — Supporting Information. [file CPDD-11-388-s001.docx]

**Figure S1.** Individual plasma concentration-time profiles for (A) digoxin after a single oral dose of 0.5 mg digoxin alone (black lines) and (B) in combination with 50 mg OM MR (gray lines), and (C) OM after a single oral dose of 50 mg OM MR alone (maroon lines) and (D) in combination with 600 mg amiodarone (blue lines). Graphs are in linear scale and inlet graphs are in semi-logarithmic scale. MR modified release; OM omecamtiv Mecarbil; SD standard deviation.
